# Association of Prophylactic Abdominal Drain Placement With Short‐Term Postoperative Outcomes After Hepatectomy: A Nationwide Inpatient Database Study in Japan

**DOI:** 10.1002/ags3.70299

**Published:** 2026-10-19

**Authors:** Keita Kouzu, Shotaro Aso, Yuki Hirano, Hiroki Matsui, Kensuke Yoshimura, Hiroki Kitagawa, Toru Kato, Naoki Yamane, Osahiko Hagiwara, Norikatsu Miyoshi, Satoru Matsuda, Hiroshi Maruyama, Keita Morikane, Junichi Sasaki, Hideo Yasunaga, Yuko Kitagawa, Manabu Watanabe, Hironori Tsujimoto

**Affiliations:** ^1^ Department of Surgery National Defense Medical College Saitama Japan; ^2^ Department of Health Services Research, Graduate School of Medicine The University of Tokyo Tokyo Japan; ^3^ Department of Hepatobiliary–Pancreatic and Gastrointestinal Surgery International University of Health and Welfare School of Medicine Chiba Japan; ^4^ Department of Clinical Epidemiology and Health Economics, School of Public Health The University of Tokyo Tokyo Japan; ^5^ Center for Next Generation of Community Health Chiba University Hospital Chiba Japan; ^6^ Department of Infectious Diseases Hiroshima University Hospital Hiroshima Japan; ^7^ Department of Surgery, Division of Gastroenterological Surgery Sapporo Medical University Sapporo Japan; ^8^ Department of Cardiovascular Surgery Kawasaki Medical School Okayama Japan; ^9^ Department of Surgery Toho University Ohashi Medical Center Tokyo Japan; ^10^ Department of Gastroenterological Surgery, Graduate School of Medicine The University of Osaka Osaka Japan; ^11^ Department of Surgery Keio University School of Medicine Tokyo Japan; ^12^ Department of Surgery Nippon Medical School Tama Nagayama Hospital Tokyo Japan; ^13^ Division of Clinical Laboratory and Infection Control Yamagata University Hospital Yamagata Japan; ^14^ Department of Emergency and Critical Care Medicine Keio University School of Medicine Tokyo Japan

## Abstract

**Aim:**

Although randomized trials have discouraged routine prophylactic abdominal drainage after uncomplicated hepatectomy, these drains are still widely used in clinical practice. This study evaluated the association between prophylactic abdominal drain placement and short‐term outcomes after hepatic resection using a nationwide inpatient database.

**Methods:**

We identified patients who underwent initial hepatic resection for liver tumors from the Japanese Diagnosis Procedure Combination database. Patients were classified into drain and no‐drain groups according to whether a prophylactic abdominal drain was placed intraoperatively. In‐hospital outcomes were compared using propensity score overlap weighting. Subgroup analyses were performed for intra‐abdominal infection and in‐hospital mortality.

**Results:**

Among 73 613 eligible patients, 62 934 (85.5%) received prophylactic abdominal drains. After overlap weighting, the incidence of intra‐abdominal infection did not differ significantly between the drain and no‐drain groups (5.1% vs. 5.5%; risk difference, −0.3%; 95% confidence interval [CI], −0.8 to 0.1). Bile leakage, postoperative transfusion, postoperative drainage, and hospitalization costs were also comparable between groups. In contrast, prophylactic drain placement was associated with lower in‐hospital mortality (0.5% vs. 1.0%; risk difference, −0.6%; 95% CI, −0.8 to −0.4). Subgroup analyses showed that this association was more evident in patients with low body mass index and those treated at low‐ or middle‐volume hospitals.

**Conclusion:**

Prophylactic abdominal drain placement after hepatic resection was not associated with a lower incidence of intra‐abdominal infection.

## 1 Introduction

Hepatic resection is widely performed as curative treatment for primary and metastatic tumors. Although advances in surgical techniques and perioperative management have improved the short‐term outcomes of hepatectomy [1], organ/space surgical site infection (SSI) reportedly occurs in 5.0% of patients undergoing hepatectomy, with an in‐hospital mortality rate of 1.7% [2]. Postoperative complications not only increase hospital length of stay and healthcare costs but are also associated with impaired long‐term survival in patients with malignancy [3].

Enhanced recovery after surgery protocols strongly recommend against routine prophylactic abdominal drain placement in hepatectomy without biliary reconstruction [4]. However, prophylactic abdominal drainage has traditionally been used to facilitate early detection and management of bile leakage and postoperative hemorrhage. Meta‐analyses have shown that routine drainage does not reduce SSI incidence or mortality [5, 6]. Moreover, a recent large‐scale randomized controlled trial reported that the incidence of bile leakage and Clavien–Dindo grade ≥ 3 complications was significantly higher in patients with drains compared to those without drains [7], suggesting that unnecessary prophylactic abdominal drain placement may paradoxically increase postoperative complications.

Despite mounting evidence against routine drainage, prophylactic drains are still widely used in clinical practice. A survey of 191 hepatobiliary surgeons worldwide indicated that 58.6% routinely placed drains after hepatectomy [8, 9]. This discrepancy between existing evidence and actual clinical practice raises the clinically relevant question of whether the association between prophylactic drain placement and postoperative outcomes differs according to patient‐ and institution‐level factors.

The present study aimed to investigate the association between prophylactic abdominal drain placement and the short‐term postoperative outcomes after hepatic resection, thereby providing evidence to inform optimal drain use in routine clinical practice, using a nationwide Japanese inpatient database.

## 2 Material and Methods

### 2.1 Ethics

The requirement for informed consent was waived because anonymized data were used in this study. The study protocol was approved by the Institutional Review Board of the University of Tokyo [approval number: 3501‐(5)].

### 2.2 Data Source

This retrospective cohort study analyzed data extracted from the Diagnosis Procedure Combination (DPC), a comprehensive nationwide inpatient database in Japan. As of April 2024, more than 1200 acute‐care hospitals, including all 82 university hospitals, participated in the database. Approximately 44% of the hospitals accredited by the Japan Surgical Society are registered in the database [10].

The database contains demographic information (e.g., age, sex), diagnostic details (e.g., diagnoses, comorbidities at admission, and complications after admission), and clinical data (e.g., body weight, height, smoking status, and Barthel Index). Administrative claims data related to anesthesia, surgical procedures, and medications are also included. Diagnoses and complications are recorded according to the International Classification of Diseases, 10th Revision (ICD‐10), whereas surgical procedures and medications are coded in Japanese‐specific formats. The DPC system functions under a per diem bundled‐payment structure, which mandates that hospitals maintain highly accurate documentation. Previous validation studies have demonstrated a sensitivity of 50%–80% and specificity of 96% for recorded primary diagnoses, while both the sensitivity and specificity for recorded surgical procedures exceed 90% [11].

### 2.3 Study Protocol

We identified patients aged 18 years or above who underwent initial hepatic resection without biliary reconstruction for liver tumors between January 2014 and March 2022. Patients with missing data in the fiscal year of surgery were excluded. Patients who underwent prophylactic abdominal drain placement on the day of hepatic resection were assigned to the drain group, whereas those who did not undergo drain placement were assigned to the no‐drain group (Figure S1).

### 2.4 Outcomes and Covariates

The primary endpoint was the incidence of postoperative intra‐abdominal infection (organ/space SSI, defined as bile leakage, intra‐abdominal abscess, or pancreatic fistula). The secondary outcomes included in‐hospital mortality, bile leakage, postoperative blood transfusion, postoperative ventilation ≥ 2 days, postoperative drainage, length of postoperative hospital stay, and total hospitalization costs (converted to USD at an exchange rate of 156.1 JPY/USD as of February 27, 2026). Table S1 provides the ICD and Japanese procedural codes corresponding to these outcomes.

The study covariates were age, sex, body mass index (BMI), smoking history, Barthel Index, Charlson Comorbidity Index, hypertension, diabetes, chronic obstructive pulmonary disease, renal failure, liver disease, preoperative corticosteroid use, preoperative transfusion, tumor category (primary liver malignancy or other eligible liver tumor), extent of hepatic resection (major or minor hepatectomy), surgical approach (open or minimally invasive), anesthesia time, intraoperative transfusion, hospital type (teaching or non‐teaching), hospital hepatectomy volume, and fiscal year. BMI was classified into four categories: < 18.5, 18.5–24.9, 25.0–29.9, and ≥ 30.0 kg/m^2^. A Barthel Index of < 85 indicated impairment in the activities of daily living. The Charlson Comorbidity Index was calculated based on the ICD‐10 codes and grouped as 2, 3, 4, or ≥ 5. The extent of hepatic resection was categorized into major and minor hepatectomies. In accordance with previous studies, major hepatectomy was defined as the resection of three or more Couinaud segments [7]. Minimally invasive surgery encompassed both laparoscopic and robot‐assisted procedures. We categorized hospital volume into tertiles based on the annual hospital hepatectomy volume: low (< 13.1), middle (13.1–29.9), and high (≥ 29.9).

### 2.5 Statistical Analysis

To address potential confounding, we conducted propensity score overlap weighting to compare the postoperative outcomes between the drain and no‐drain groups. The propensity score model included the following covariates: age, sex, BMI, smoking history, Barthel Index, Charlson Comorbidity Index, hypertension, diabetes, chronic obstructive pulmonary disease, renal failure, liver disease, preoperative corticosteroid use, preoperative transfusion, tumor category (primary liver malignancy or other eligible liver tumor), extent of hepatic resection (major or minor hepatectomy), surgical approach (open or minimally invasive), anesthesia time, intraoperative transfusion, hospital type (teaching or non‐teaching), hospital hepatectomy volume, and fiscal year. In the overlap weighting framework, each individual received a weight that was inversely proportional to the likelihood of being assigned to the treatment group. That is, patients with drain placement were weighted by 1 minus the propensity score, whereas those without drain placement were weighted by the propensity score. Covariate balance was evaluated using absolute standardized differences, with values < 0.10 deemed acceptable.

Subgroup analyses were performed based on the BMI category, extent of hepatic resection, surgical approach, and hospital volume.

We performed a sensitivity analysis using generalized estimating equations to account for within‐hospital clustering associated with differences in hospital‐level practice patterns and perioperative management systems. After adjustment for the same patient characteristics and perioperative factors as those in the primary analysis, odds ratios and 95% confidence intervals (CI) were estimated for the associations of drain placement with intra‐abdominal infection and in‐hospital mortality.

Continuous variables were compared using Student's *t*‐test, and categorical variables were compared using the chi‐squared test. Statistical significance was defined as a two‐sided *p*‐value of < 0.05. All analyses were conducted using STATA version 19.5 (StataCorp LLC, College Station, TX, USA).

## 3 Results

A total of 73 613 patients were included in this study, of whom 62 934 (85.5%) underwent prophylactic intra‐abdominal drain placement during surgery. Table 1 summarizes the patients' baseline characteristics before and after overlap weighting. The proportion of major hepatectomies was 19.1% in the overall cohort. Prophylactic intra‐abdominal drains were placed in 91.6% of major hepatectomies and 84.1% of minor hepatectomies. Prophylactic intra‐abdominal drains were inserted in 77.5% of minimally invasive hepatectomies and in 89.8% of open hepatectomies. The proportion of patients who underwent major hepatectomy was higher in the drain group than in the no‐drain group (20.5% vs. 11.1%, respectively). The proportion of minimally invasive surgery was lower in the drain group than that in the no‐drain group (31.7% vs. 54.0%). The prevalence of liver disease did not differ significantly between the two groups.

**TABLE 1:** Patient background characteristics.

Variables	Before overlap weighting			After overlap weighting		
	No‐drain group	Drain group	ASD	No‐drain group	Drain group	ASD
	*n* = 10 679	*n* = 62 934				
Age, years (SD)	68.4 (11.7)	69.0 (11.0)	0.05	68.5 (11.5)	68.5 (11.4)	< 0.01
Male, *n* (%)	6842 (64.1)	42 552 (67.6)	0.07	(64.9)	(64.9)	< 0.01
Body mass index, kg/m^2^, *n* (%)			0.02			< 0.01
< 18.5	894 (8.4)	4905 (7.8)		(8.3)	(8.3)	
18.5–24.9	6631 (62.6)	39 467 (63.0)		(62.7)	(62.7)	
25.0–29.9	2570 (24.2)	15 357 (24.5)		(24.3)	(24.3)	
≥ 30.0	503 (4.7)	2888 (4.6)		(4.7)	(4.7)	
Smoking, *n* (%)	5825 (54.5)	34 270 (54.5)	0.00	(54.4)	(54.4)	< 0.01
Impaired activities of daily living, *n* (%)	313 (3.0)	1677 (2.7)	0.02	(2.9)	(2.9)	< 0.01
Charlson comorbidity index, *n* (%)			0.05			< 0.01
2	4131 (38.7)	23 189 (36.8)		(38.3)	(38.3)	
3	395 (3.7)	2360 (3.7)		(3.7)	(3.7)	
4	4329 (40.5)	25 626 (40.7)		(40.7)	(40.7)	
≥ 5	1824 (17.1)	11 759 (18.7)		(17.4)	(17.4)	
Hypertension	3052 (28.6)	19 733 (31.4)	0.06	(29.2)	(29.2)	< 0.01
Diabetes mellitus	2913 (27.3)	18 511 (29.4)	0.05	(27.8)	(27.8)	< 0.01
Chronic obstructive pulmonary disease	152 (1.4)	825 (1.3)	0.01	(1.4)	(1.4)	< 0.01
Renal failure	263 (2.5)	1480 (2.4)	0.01	(2.4)	(2.4)	< 0.01
Liver disease	836 (7.8)	4729 (7.5)	0.01	(7.9)	(7.9)	< 0.01
Corticosteroid use before surgery	166 (1.6)	886 (1.4)	0.01	(1.5)	(1.5)	< 0.01
Preoperative transfusion, *n* (%)						
Fresh frozen plasma	30 (0.3)	86 (0.1)	0.03	(0.2)	(0.2)	< 0.01
Red blood cell	119 (1.1)	641 (1.0)	0.01	(1.0)	(1.0)	< 0.01
Platelet concentrates	42 (0.4)	152 (0.2)	0.03	(0.3)	(0.3)	< 0.01
Malignant, *n (%)*	6413 (60.1)	40 737 (64.7)	0.10	(61.3)	(61.3)	< 0.01
Major hepatectomy, *n* (%)	1186 (11.1)	12 907 (20.5)	0.26	(12.6)	(12.6)	< 0.01
Minimally invasive approach, *n* (%)	5767 (54.0)	19 920 (31.7)	0.46	(49.6)	(49.6)	< 0.01
Duration of anesthesia, min (SD)	317.8 (114.6)	368.3 (109.6)	0.45	327.4 (115.4)	327.4 (104.1)	< 0.01
Transfusion on the day of surgery, *n* (%)						
Fresh frozen plasma	666 (6.2)	7701 (12.2)	0.21	(7.0)	(7.0)	< 0.01
Red blood cell	743 (7.0)	9053 (14.4)	0.24	(7.9)	(7.9)	< 0.01
Platelet concentration	295 (2.8)	2108 (3.3)	0.03	(2.8)	(2.8)	< 0.01
Teaching hospital, *n* (%)	8649 (81.0)	47 767 (75.9)	0.12	(80.1)	(80.1)	< 0.01
Hospital volume, high, *n* (%)			0.16			< 0.01
Low	2794 (26.2)	20 598 (32.7)		(27.2)	(27.2)	
Middle	3639 (34.1)	21 230 (33.7)		(34.6)	(34.6)	
High	4246 (39.8)	21 106 (33.5)		(38.2)	(38.2)	
Fiscal year, *n* (%)			0.14			< 0.01
2014	1155 (10.8)	7656 (12.2)		(11.2)	(11.2)	
2015	1084 (10.2)	7455 (11.8)		(10.6)	(10.6)	
2016	1124 (10.5)	7768 (12.3)		(10.8)	(10.8)	
2017	1158 (10.8)	7342 (11.7)		(11.0)	(11.0)	
2018	1163 (10.9)	7055 (11.2)		(10.7)	(10.7)	
2019	1165 (10.9)	6962 (11.1)		(10.9)	(10.9)	
2020	1275 (11.9)	6805 (10.8)		(11.7)	(11.7)	
2021	1310 (12.3)	6128 (9.7)		(11.9)	(11.9)	
2022	1245 (11.7)	5763 (9.2)		(11.2)	(11.2)	

Abbreviations: ASD, absolute standardized difference; SD, standard deviation.

After overlap weighting, all covariates appeared well balanced. Table 2 presents the primary and secondary outcomes before and after the overlap weighting. After overlap weighting, no significant difference was apparent in the proportion of intra‐abdominal infections between the groups [5.1% vs. 5.5%; risk difference (RD), −0.3%; 95% CI, −0.8 to 0.1]. The in‐hospital mortality was significantly lower in the drain group than in the no‐drain group (0.5% vs. 1.0%; RD, −0.6%; 95% CI, −0.8 to −0.4). The incidence of bile leakage, postoperative transfusion, postoperative ventilation, postoperative drainage, and total hospitalization costs did not differ significantly between the two groups. Prophylactic drain placement was associated with a prolonged postoperative length of stay (RD, 0.3 days; 95% CI: 0.01–0.7). The generalized estimating equations analysis accounting for hospital‐level clustering included 72 071 patients from 1147 hospitals. After accounting for within‐hospital correlation in patient outcomes, drain placement was not significantly associated with intra‐abdominal infection (odds ratio, 0.96; 95% CI, 0.87–1.06). In contrast, drain placement was associated with lower in‐hospital mortality (odds ratio, 0.50; 95% CI, 0.39–0.63).

**TABLE 2:** Postoperative outcomes before and after overlap weighting.

Outcomes	Before overlap weighting		After overlap weighting				
	No‐drain group	Drain group	No‐drain group	Drain group			
	*n* = 10 679	*n* = 62 934			Risk difference	95% confidence interval	*p*
Intra‐abdominal infections, *n* (%)	564 (5.3)	4038 (6.4)	(5.5)	(5.1)	−0.3	−0.8 to 0.1	0.17
Death, *n* (%)	108 (1.0)	470 (0.8)	(1.0)	(0.5)	−0.6	−0.8 to −0.4	< 0.01
Bile leakage, *n* (%)	84 (0.8)	972 (1.5)	(0.9)	(1.0)	0.1	−0.07 to 0.3	0.21
Postoperative transfusion, *n* (%)	410 (3.8)	3556 (5.7)	(4.2)	(4.1)	−0.03	−0.4 to 0.4	0.89
Postoperative ventilation, *n* (%)	11 (0.1)	102 (0.2)	(0.1)	(0.1)	0.02	−0.05 to 0.09	0.56
Postoperative drainage, *n* (%)	102 (1.0)	902 (1.4)	(1.1)	(1.0)	−0.04	−0.3 to 0.2	0.71
					Difference		
Length of postoperative hospital stay, days (SD)	13.3 (15.1)	16.2 (18.1)	13.8 (15.4)	14.1 (14.6)	0.3	0.008–0.7	0.045
Total hospitalization costs, US$ (SD)	10,684 (7022)	11,751 (6989)	10,848 (7178)	10,859 (5979)	−11	−141 to 165	0.88

Abbreviation: SD, standard deviation.

Figure 1 illustrates the subgroup analyses of intra‐abdominal infections. Among patients treated in high‐volume hospitals, the proportion of intra‐abdominal infections was significantly lower in the drain group than in the no‐drain group. The incidence of intra‐abdominal infections did not differ significantly between the two groups in the other subgroup categories.

**FIGURE 1:**
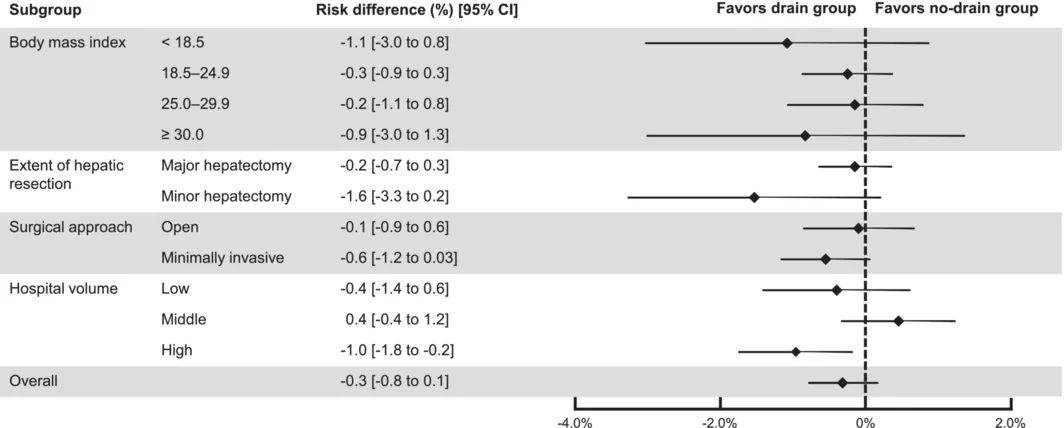
Subgroup analyses of intra‐abdominal infection after hepatic resection according to prophylactic abdominal drain placement.

Figure 2 shows the subgroup analysis of in‐hospital mortality. Subgroup analysis revealed that mortality was significantly lower in the drain group than in the no‐drain group among patients with BMI < 18.5 kg/m^2^ and 18.5–24.9 kg/m^2^, whereas no significant differences were observed between the two groups in those with BMI 25.0–29.9 kg/m^2^ and ≥ 30.0 kg/m^2^. Regarding the mortality rate, it was significantly lower in the drain group than that in the no‐drain group based on the extent of hepatic resection and surgical approach. Mortality was significantly lower in the drain group than in the no‐drain group among patients treated in low‐ and middle‐volume hospitals, while no significant difference was observed between the two groups in patients treated in high‐volume hospitals.

**FIGURE 2:**
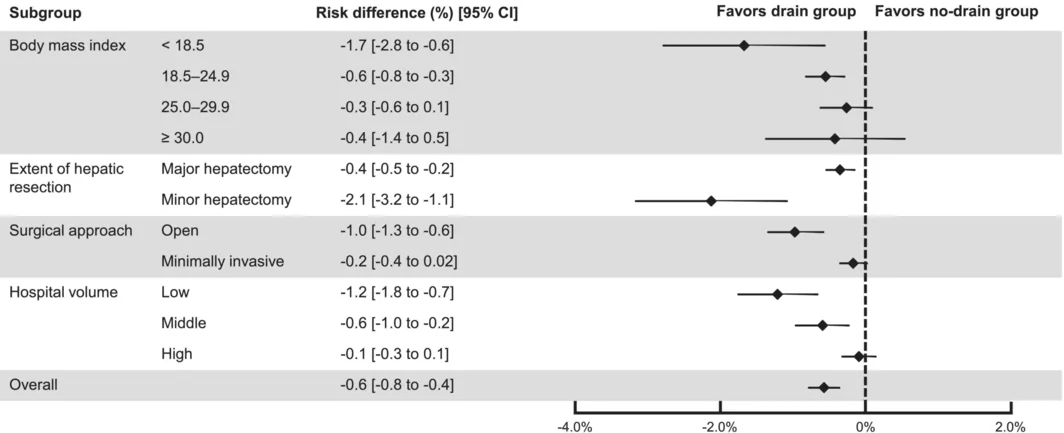
Subgroup analyses of in‐hospital mortality after hepatic resection according to prophylactic abdominal drain placement. CI, confidence interval.

## 4 Discussion

This study investigated the association between prophylactic abdominal drain placement and the short‐term outcomes after hepatic resection using a national inpatient database. Our findings demonstrated that prophylactic drain placement was not associated with lower incidence of intra‐abdominal infection but was significantly associated with lower in‐hospital mortality.

The lack of a significant association between prophylactic drain placement and intra‐abdominal infection or bile leakage was also observed in prior randomized controlled trials and meta‐analyses [4, 5, 6]. In our exploratory subgroup analysis based on hospital volume, the proportion of intra‐abdominal infections was significantly lower in the drain group than in the no‐drain group among patients treated in high‐volume hospitals, whereas no significant difference was evident between drain and non‐drain use in low‐ or middle‐volume hospitals. One possible explanation for this finding is that high‐volume hospitals may have better established postoperative management systems, enabling them to appropriately interpret and utilize information obtained from prophylactic abdominal drains. In addition, surgeons at high‐volume centers may make more selective decisions regarding drain placement based on the intraoperative findings and patient risk profiles.

In the present study, prophylactic intra‐abdominal drain placement was not associated with a reduction in the incidence of intra‐abdominal infections; however, it was significantly associated with lower in‐hospital mortality. One possible reason is that prophylactic drains do not prevent the occurrence of complications per se, but rather facilitate the monitoring of postoperative complications and enable earlier intervention. Bile leakage can progress to peritonitis or intra‐abdominal sepsis, and some refractory cases may require additional surgical intervention, underscoring the importance of prompt and accurate diagnosis [12]. No significant difference was observed between the drain and no‐drain groups among patients treated at high‐volume hospitals, whereas mortality was significantly lower in the drain group among those treated at low‐ and middle‐volume hospitals. One possible interpretation is that, in high‐volume hospitals, perioperative management is generally well‐standardized and baseline mortality is low, thereby limiting any additional survival benefit associated with drain placement. In contrast, in low‐ and middle‐volume hospitals, where resources and experience related to postoperative monitoring may vary more substantially, drain placement may provide additional clinical information that facilitates the identification of severe complications and may thereby contribute to lower in‐hospital mortality. However, in the present study, significant differences in bile leakage, postoperative drainage, transfusion, or prolonged ventilation were not observed between the drain and no‐drain groups. Furthermore, we could not identify the specific causes of death in the DPC database. Hospital volume may not adequately capture differences in institutional policies, postoperative management, and rescue systems. Therefore, our study cannot determine whether the significant association between drain placement and lower in‐hospital mortality is clinically meaningful.

The estimates derived in the present study primarily apply to the overlap population, in whom either drainage strategy was more plausible based on measured characteristics. This population is clinically relevant to the unresolved question of selective drain use. However, our findings may not be generalizable to patients in whom the indication for drain placement would be almost certain, including highly complex surgical cases.

This study had some limitations. First, we were unable to obtain information on the severity of bile leakage from our database. However, no significant between‐group differences were observed in postoperative percutaneous drainage. Therefore, the inability to determine the severity of the complications is unlikely to have affected the principal conclusions of this study. Second, we were unable to determine the reasons for drain placement or the decision‐making processes of individual surgeons. Although routine prophylactic drainage was widely used in this cohort despite existing evidence and guideline recommendations, the present study could not directly determine why this practice persists. In addition, drain placement may have been influenced by institutional policies and practice patterns. Although the analysis adjusted for hospital type and hepatectomy volume, residual confounding arising from these unmeasured factors may have affected the observed results. Third, information on drain management, such as the duration and type of drain placement, was not available. Prolonged drain placement may be a potential risk factor for retrograde bacterial infection [13], and the negative pressure generated by closed‐suction drainage systems has been suggested to potentially increase the risk of bile leakage [7]. However, the aim of the present study was to investigate the association of prophylactic abdominal drains with the outcomes, rather than subtle differences in drain management. Fourth, the DPC database does not contain information on pain, discomfort, delayed mobilization, cosmetic impairment, or injury to vessels or organs during drain insertion. Fifth, the DPC codes and algorithms used in this study to identify bile leakage, intra‐abdominal abscess, and other postoperative intra‐abdominal infections have not been individually validated against medical records. Although diagnostic information recorded in the DPC database has generally been reported to have high specificity, its sensitivity varies depending on the diagnosis. Therefore, the possibility of outcome misclassification, particularly due to underestimation of complications, cannot be excluded. Sixth, because the DPC database did not provide the exact intraoperative timing of drain placement, its temporal relationship with anesthesia duration and intraoperative transfusion could not be determined. Although these variables were included to account for operative complexity, they may have reflected factors occurring close to the decision regarding drain placement. The possibility of residual bias remains, and the observed association with mortality should not be interpreted as evidence of a causal effect of prophylactic drainage.

In conclusion, prophylactic intra‐abdominal drain placement after hepatic resection was not associated with a lower incidence of intra‐abdominal infections. The effect of prophylactic drain placement may vary according to patient characteristics and institutional factors, and its routine use is not recommended universally. Further prospective studies are required to clarify the role of prophylactic drain placement in specific patient populations.

## Author Contributions

**Keita Kouzu:** data curation, conceptualization, formal analysis, investigation, methodology, validation, visualization, writing – original draft. **Shotaro Aso:** conceptualization, data curation, formal analysis, investigation, visualization, methodology, resources, supervision, validation. **Yuki Hirano:** conceptualization, data curation, investigation, methodology, project administration, supervision, validation. **Hiroki Matsui:** data curation, resources. **Kensuke Yoshimura:** data curation, resources. **Hiroki Kitagawa:** conceptualization, methodology. **Toru Kato:** conceptualization, methodology. **Naoki Yamane:** conceptualization, methodology. **Osahiko Hagiwara:** conceptualization, methodology. **Norikatsu Miyoshi:** conceptualization, methodology. **Satoru Matsuda:** conceptualization, methodology. **Hiroshi Maruyama:** conceptualization, methodology. **Keita Morikane:** conceptualization, methodology. **Junichi Sasaki:** conceptualization, funding acquisition, project administration, resources, supervision. **Hideo Yasunaga:** conceptualization, methodology, project administration, resources, supervision. **Yuko Kitagawa:** conceptualization, project administration, resources, supervision. **Manabu Watanabe:** conceptualization, methodology. **Hironori Tsujimoto:** conceptualization, supervision, writing – review and editing, validation.

## Funding

This work was supported by grants conferred by the Ministry of Health, Labour and Welfare, Japan (26AA2006 and 26AA2001).

## Ethics Statement

The requirement for informed consent was waived because anonymized data were used in this study. The study protocol was approved by the Institutional Review Board of the University of Tokyo [approval number: 3501‐(5)].

## Conflicts of Interest

Yuko Kitagawa was the recipient of research grants conferred to his institution by Terumo Corporation. The other authors declare no conflicts of interest.

## Supporting information


**Figure S1:** Participant flow diagram.

**Table S1:** Definitions of surgical procedures, comorbidities, and postoperative complications.

## Data Availability

The data that support the findings of this study are not publicly available.
