# Supplementary material for: Association of Prophylactic Abdominal Drain Placement With Short‐Term Postoperative Outcomes After Hepatectomy: A Nationwide Inpatient Database Study in Japan
Source: Ann Gastroenterol Surg. 2026 Oct 19:10.1002/ags3.70299. Online ahead of print. doi: 10.1002/ags3.70299 (PMC13680735; doi:10.1002/ags3.70299)
Supplement: Supplementary file 1 [file AGS3-9999-0-s001.docx]

**Supplemental Table 1. Definitions of surgical procedures, comorbidities, and postoperative complications**

|  | **ICD-10 codes** | **Original Japanese codes or procedures** |
| --- | --- | --- |
| Biliary fistula | K833 |  |
| Intra-abdominal infections | K650, K833, K868, K918, T813, T814 |  |
| Postoperative mechanical ventilation |  | Mechanical ventilation use lasting >2 days following surgery |
| Postoperative drainage |  | J0021, J0022, J021, K637-2  The devices required for percutaneous drainage were billed on the day after surgery |
| Malignant |  | C220, C221, C222, C223, C224 |
| Major hepatectomy |  | K695-5, K695-6, K695-7, K695-2-5, K695-2-6 |
| Minor hepatectomy |  | K695-1, K695-2, K695-3, K695-4, K695-2-1, K695-2-2, K695-2-3, K695-2-4 |
| Hypertension | I10–I15 |  |
| Diabetes mellitus | E10–E14 |  |
| Chronic obstructive pulmonary disease | J44 |  |
| Renal failure | N18 |  |
| Liver disease | K702, K703, K704, K711, K717, K720, K721, K729, K740, K741, K743, K744, K745, K746, K765, K766, K767 |  |

ICD-10, International Classification of Diseases, Tenth Revision
